# Identifying reactive intermediates by mass spectrometry

**DOI:** 10.1039/d0sc04754f

**Published:** 2020-10-20

**Authors:** Jaya Mehara, Jana Roithová

**Affiliations:** Institute for Molecules and Materials, Radboud University Heyendaalseweg 135 6525 AJ Nijmegen The Netherlands jana.roithova@ru.nl

## Abstract

Development of new reactions requires finding and understanding of novel reaction pathways. In challenging reactions such as C–H activations, these pathways often involve highly reactive intermediates which are the key to our understanding, but difficult to study. Mass spectrometry has a unique sensitivity for detecting low abundant charged species; therefore it is increasingly used for detection of such intermediates in metal catalysed- and organometallic reactions. This perspective shows recent developments in the field of mass spectrometric research of reaction mechanisms with a special focus on going beyond mass-detection. Chapters discuss the advantages of collision-induced dissociation, ion mobility and ion spectroscopy for characterization of structures of the detected intermediates. In addition, we discuss the relationship between the condensed phase chemistry and mass spectrometric detection of species from solution.

## Abstract 2

Modern approaches of mass spectrometry can identify reaction intermediates and provide a unique insight into their structure, properties and kinetics.

## Graphical abstract

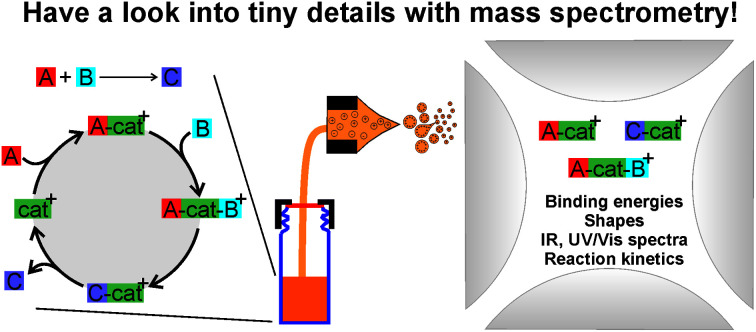

## Introduction

Progress in chemistry is driven by findings of new chemical transformations that allow us to construct new molecules and materials. The further advances are based on our understanding of the chemical reactions and on our ability to use this knowledge to control the reactivity. Hence, development of new reactions goes always hand in hand with mechanistic studies that rationalize these reactions and give us a framework to further build up on them.

Many spectroscopic methods are used to monitor chemical reactions. The *in situ* methods such as optical spectroscopies (IR or UV-Vis spectroscopy) or NMR spectroscopy can determine rates of transformation of reactants to the products and in connection with modern physical organic chemistry methods provide deep insight into reaction mechanisms.^1–3^ Detection of key reaction intermediates is another important part in elucidation of reaction mechanisms. It can be easy for some reactions, but it could be rather challenging for other ones.^4^ Especially, in recent organometallic, photocatalytic, electrocatalytic or other catalytic reactions that are based on generation of highly reactive species in solution, detection of intermediates can be challenging.

Reactive intermediates are often short-lived and their concentration is small. Hence, *in situ* detection of these species often relies on their unique properties that help in suppressing the signals of dominant species in solution. An example could be EPR (electron paramagnetic resonance) spectroscopy of radical intermediates or NMR spectroscopy based on elements present just in a catalyst and its complexes.

Another strategy relies on *ex situ* approaches. The popular *ex situ* approach is crystallization of a metal-complex intermediate out of the reaction mixture. X-ray crystallography offers many details about these intermediates and therefore provides valuable data. However, it also has pitfalls. Firstly, the effort to crystalize the intended reactive intermediates is often accompanied by modifications of the complexes in order to make them more stable which can introduce a bias to the study. Secondly, crystallization may yield off-cycle species or resting states rather than the reactive intermediates.

Mass spectrometry analysis is another *ex situ* approach. It is extremely sensitive and has a large dynamic range and thus allows detection of minor species. The reaction mixtures can be directly analysed using electrospray ionization interface.^5^ The simple implementation and a broad availability of mass spectrometers resulted in growing popularity of mass spectrometry analysis in investigation of reaction mechanisms. Particularly, catalytic organometallic reactions are often addressed by mass spectrometry, because metal-containing intermediates are usually easily ionized (or present as ions in solution *per se*) and thus selectively detected.^6,7^

Mass spectrometry analysis of reaction intermediates has also its drawbacks. Unlike NMR or IR spectroscopies, it does not provide direct structural information. Mass spectrometry analysis provides direct information only about the elemental composition. However, many methods were developed that overcome this problem and we will discuss them in this perspective.^8^ Another, more pressing problem is that electrospray ionization can generate ions that are not relevant for the processes in solution. Blind assignment of the detected ions to the reactive intermediates in solution can lead to significant misinterpretations of reaction mechanisms. Therefore, we will also address this aspect here.

### Detection of reactive intermediates by ESI-MS

Mass spectrometry detects ions. Hence, reactions making use of charged intermediates are easily monitored by electrospray ionization mass spectrometry.^5,9–11^ Typically, such intermediates are operative in reactions catalysed by cationic complexes of gold, silver, copper, rhodium or ruthenium with non-coordinating counterions such as SbF_6_^−^, BF_4_^−^, PF_6_^−^, *etc.* Other examples are organocatalytic reactions proceeding *via* iminium ions and basic intermediates. The latter can be easily detected as protonated or as sodiated ions.^12,13^

C–C coupling reactions belong to the most important chemical transformations. These reactions are typically catalysed by palladium complexes that are often neutral in solution. They can be detected as protonated ions or as anions,^14,15^ but it is by far not a general rule. Therefore, these intermediates are often studied using charge-tagging approach. Either substrate or a ligand of the catalyst are decorated by a permanently charged group placed in a position not affecting the reaction itself.^16–20^ Thereby, the intermediates can be detected as ions, yet the “neutral reaction intermediate core” is unaffected.

**Fig. 1:**
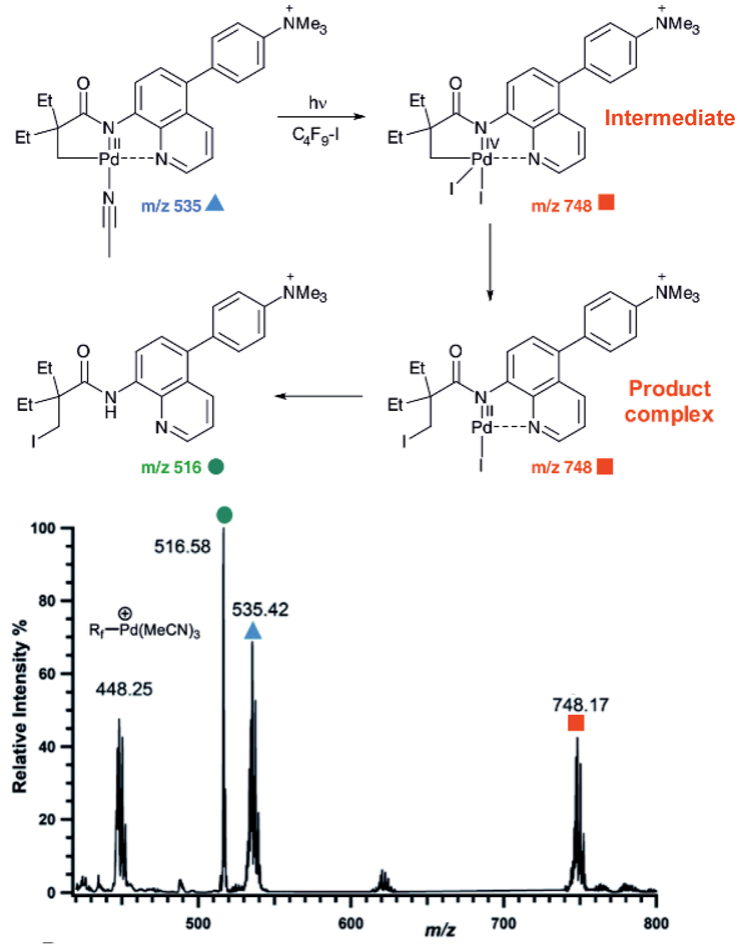
ESI-MS detection of intermediates in a palladium catalysed photochemical reaction. Adapted with changes from ref. 25 under CC BY 3.0 license.^26^

An example of using charge-tagging method could be a study of palladium catalysed C–H functionalization. Palladium catalysed C–H activation using a directing group leads to palladium(ii) intermediates such as complex **1** in Fig. 1.^21^ The activated carbon atom can be coupled with a variety of reactants.^22–24^ Czyz *et al.* explored a mechanism of the coupling with iodine promoted by photoexcitation of the palladium(ii) complex (Fig. 1).^25^ Using the charge tagging method they could monitor the starting complex (*m*/*z* 535, blue), formation of the product (*m*/*z* 516, green) and also formation of ions with *m*/*z* 748 (red) corresponding either to palladium(iv) intermediates or to palladium(ii) complex with the product.

Without going into the details of this reaction, we want to stress the common problem in the investigation of organometallic reactions nicely shown here. According to the textbook knowledge, the expected reaction intermediates and the product complexes are often connected by a reductive elimination step and thus have a different structure, but the same mass. It can be tempting to assign the detected complexes to the intermediates and thus provide the desired indices for the expected reaction mechanism. Nevertheless, it could be misleading, if the ions actually correspond to the product complexes that were formed by a different pathway. The mere observation of “the correct mass” is insufficient, instead every effort must be made to relate the mass spectra to the reaction intermediates, to the reaction mechanism and to the reaction kinetics.

### Structure of reactive intermediates by ESI-MS^2^

**Fig. 2:**
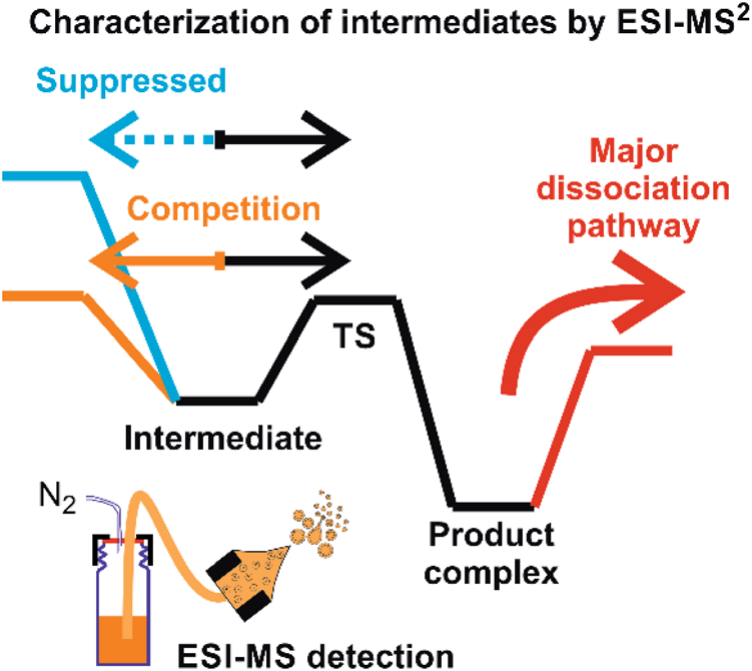
An example of a potential energy surface for gas-phase dissociation of reactive intermediates and product complexes connected by a simple reaction step such as reductive elimination (see *e.g.*Fig. 1).

Assigning possible structure to the detected ions has been classically made based on their fragmentation pattern. In detection of intermediates in organometallic reactions (such as shown in Fig. 1), this simple approach is complicated by the fact that the intermediates and the product complexes can follow the same or similar fragmentation pathways (Fig. 2). This is especially true, if the energy barrier between the intermediate and the product complex is small and the energy demands for the fragmentations are large. For example, if the energy demand for the fragmentation of the intermediate is much larger than the energy of the transition state for the rearrangement to the product complex (blue *vs.* black pathway in Fig. 2), then we will observe the same or very similar fragmentation pattern for the intermediate and for the product complex.

**Fig. 3:**
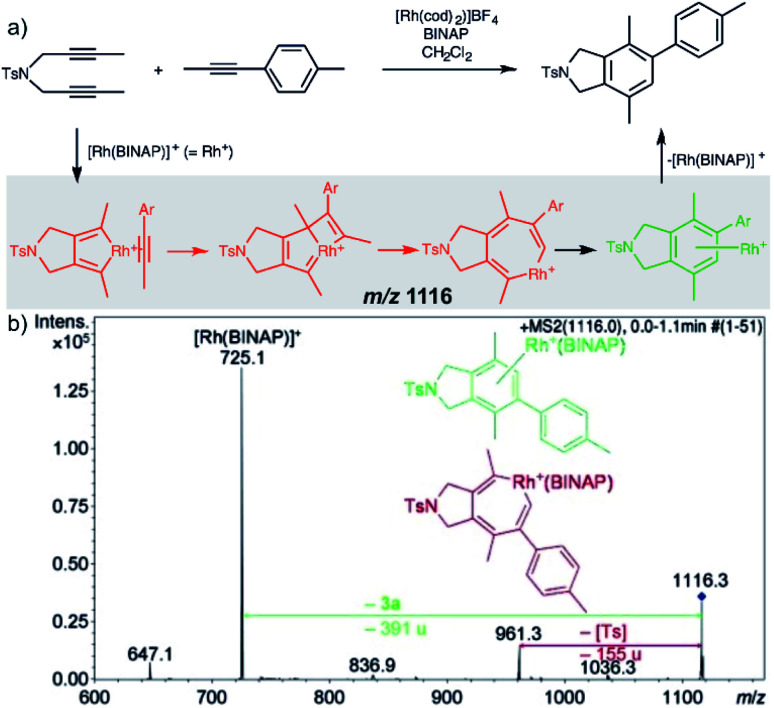
Catalytic cycle proposed for the [2+2+2] cycloaddition of diynes and monoynes under Rh/bisphosphine catalysis (the BINAP ligand has been omitted for clarity) with CID mass spectrum of the ion at *m*/*z* = 1116.3 from the reaction mixture. Adapted with permission from ref. 27. Copyright 2012 Wiley-VCH Verlag GmbH & Co. KGaA, Weinheim.

Characterization of the intermediates by ESI-MS^2^ is possible, if the fragmentation energy and the energy of the transition structure for the rearrangement to the product complex are similar (orange pathway in Fig. 2). Experimentally, this approach requires a control experiment with the complex generated from the catalyst and the independently prepared product. This is nicely shown in the work of Parera *et al.*^27^ The authors studied cyclotrimerization reaction, which is extremely demanding in terms of possible detection of isobaric complexes (Fig. 3, the grey panel). The authors detected rhodacyclic intermediate (in red, Fig. 3) and they were able to differentiate it from the product complex (in green). The reason for this successful ESI-MS^2^ characterization is that the homolytic cleavage of the tosyl group in the intermediate can kinetically compete with the reductive elimination leading to the product (Fig. 3). The product complex is dominantly losing the neutral product and does not eliminate the tosyl radical as the authors showed in a separate experiment.^27^

**Fig. 4:**
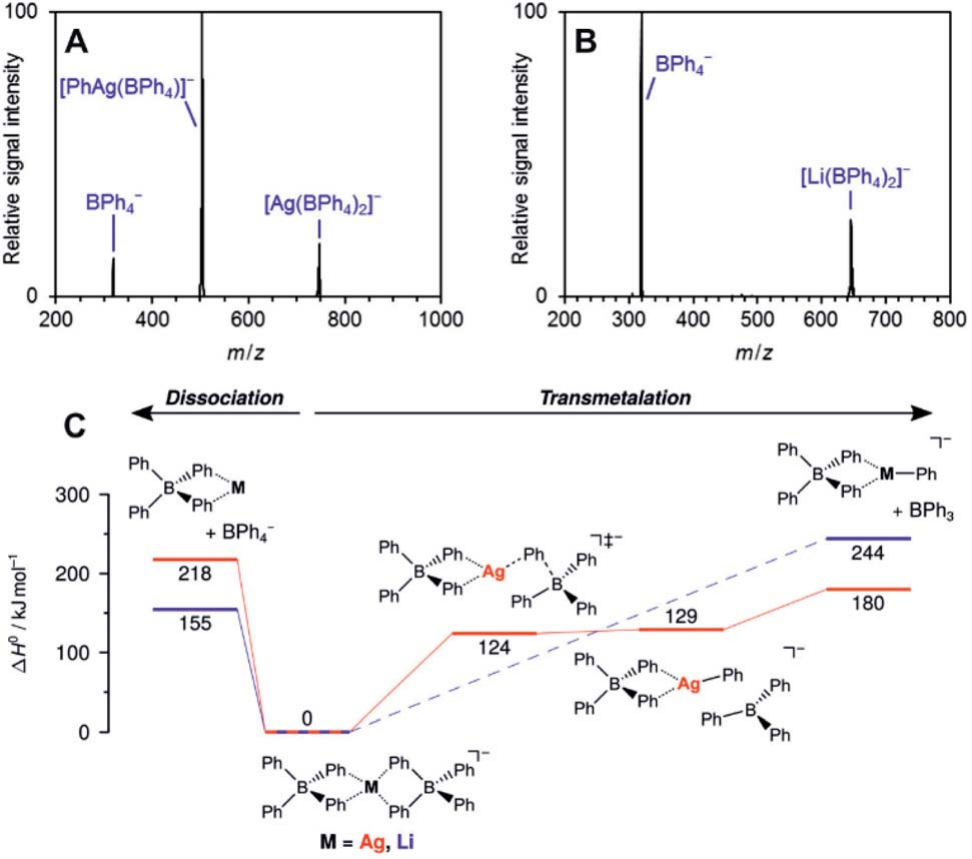
Collision induced dissociation spectra of mass-selected [M(BPh_4_)_2_]^−^ anions. (A) M = Ag, (B) M = Li. (C) Potential energy surface for dissociation of [M(BPh_4_)_2_]^−^. Adapted with permission from Ref. 28. Copyright 2020, American Chemical Society.

The work of Auth *et al.* shows how to use ESI-MS^2^ for investigation of the transformation from the reactive complex to the product.^28^ The authors studied transmetallation reaction within phenylborate complexes. They mixed Li(BPh_4_) salt with AgOTf and studied possible transfer of the phenyl group from boron to either lithium or silver. The ESI-MS detected [(BPh_4_)^−^M^+^(BPh_4_)^−^] anionic complexes (see Fig. 4 for the structure). These complexes either fragment to the [(BPh_4_)^−^M^+^] ion pair and the (BPh_4_)^−^ anion or they undergo transmetallation to yield [(BPh_4_)M(Ph)]^−^ and neutral BPh_3_. The latter is observed only for silver complexes proving that transmetallation is possible with silver, but not with lithium.

The approach of transferring complexes of this type to the gas phase and triggering the desired transformation by collisional heating offers a good control over the overall process. Hence, it creates defined conditions for systematic investigation of various metals as well as of the organic groups in transmetallation reactions. The authors demonstrated this in the study of various size metal clusters and evaluated the trends (the energy demand for the transmetallation decreases in larger clusters).

**Fig. 5:**
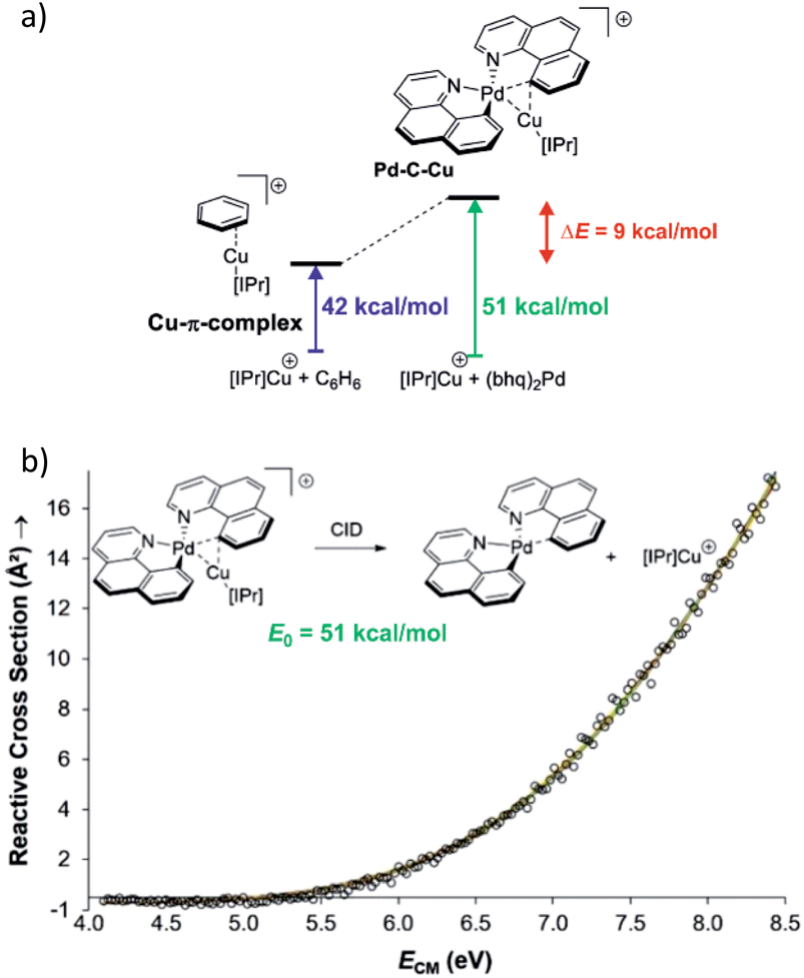
(a) Model to evaluate metal–metal interaction in a complex mimicking transmetallation transition structure. The binding energies of the ligated copper cation to benzene and to the palladium complex can be determined from energy resolved collision induced dissociation experiment. The binding energy were determined by kinetic modelling using L-CID program.^29^ (b) Zero-pressure-extrapolated cross sections (circles) with L-CID-fitted curves (lines). Inset: reaction scheme for CID of the palladium complex with the corresponding activation energy (*E*_0_). Adapted with permission from ref. 32. Copyright 2017, American Chemical Society.

Collision induced dissociation (CID) of mass-selected ions can be used also for quantitative evaluation of bond dissociation energies.^29–31^ Kinetic modelling of the energy dependent fragmentation yield in a CID experiment provides a threshold energy for the given dissociation (*e.g.*, Fig. 5b). The threshold energy is a minimum energy required for the given dissociation and thus corresponds to bond-dissociation energy. There are various experimental approaches to obtain the relevant data and there are various ways how to perform the kinetic modelling. We refer the reader to the relevant literature.^30,31^ Here, we limit the discussion to an example from the group of Peter Chen. They presented an elegant study to describe a complex that can be considered as a model for a transmetallation transition structure (see Fig. 5a). Using a thermodynamic cycle shown in Fig. 5a they could estimate stabilization of this complex by copper–palladium interaction (9 kcal mol^−1^).^32^

The approach of using tandem mass spectrometry for investigation of reaction intermediates can be extended even further. For example, Waters *et al.* used ESI-MS^*n*^ method to investigate the whole catalytic cycle of methanol oxidation by [Mo_2_O_6_(OH)]^−^ (and other metal oxides) in the gas phase.^33^ This research extends towards classical gas-phase chemistry approach for studying reactions and will not be further discussed here.^34–36^ Instead, we will concentrate on the methods providing more structural information about the reactive intermediates directly detected from solution.

### Beyond mass-analysis: ion mobility separation

Ion mobility separation is an increasingly popular addition to the mass spectrometry analysis.^37–40^ It separates isobaric ions based on their ion mobilities which essentially reflect their shapes. Clearly, this is an ideal approach to solve the dilemma of detecting reactive intermediate *vs.* the product complex discussed in the previous section. The intermediates and the product complexes have the same mass, but should have different molecular volumes and thus different ion mobility cross sections. Hence, we should be able to separate them and thus eliminate the problem of the mass overlap. The ion mobility cross section can be obtained from theoretical calculations and thus the experimental value can be compared with the predicted cross sections and assigned to a particular isomer/conformer.

**Fig. 6:**
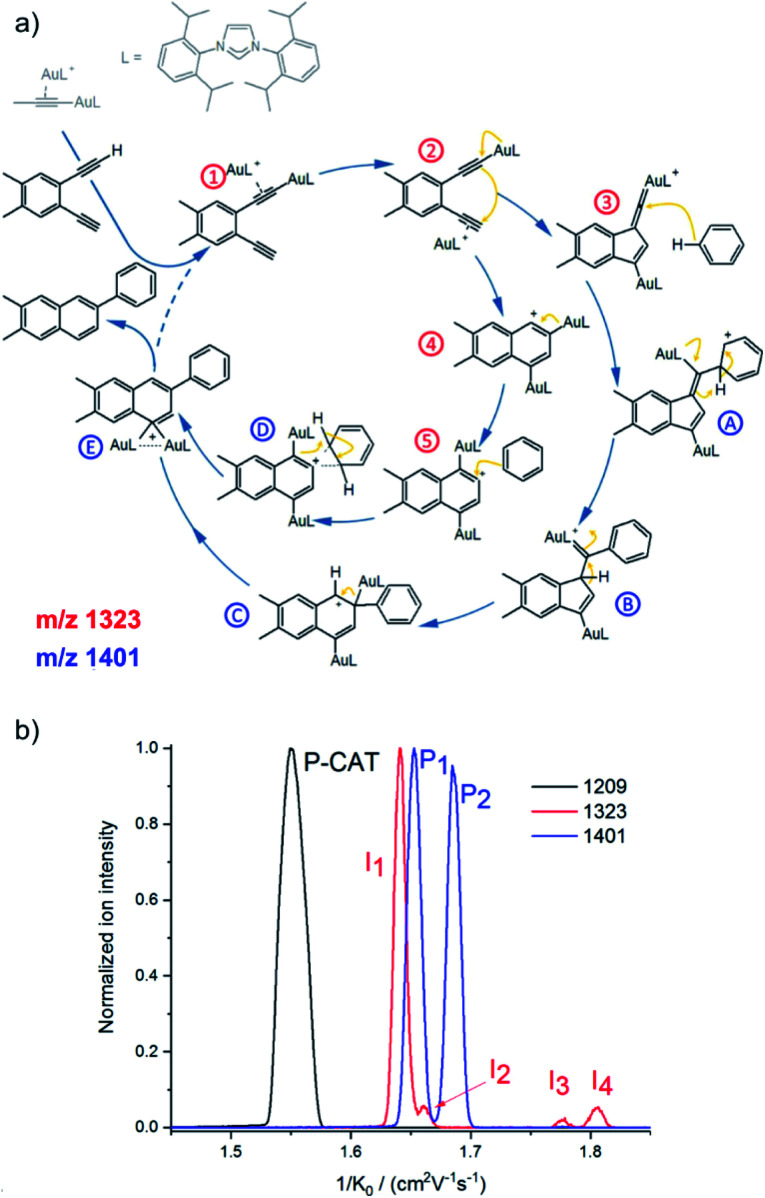
(a) Proposed mechanistic pathways for the hydroarylating aromatization of a 1,5-bis-terminal diyne R1 as catalyzed by the synergistic interplay of two gold centers [(L)Au]^+^. (b) Mobilograms: the [(L)_2_Au_2_(C_3_H_3_)]^+^ precatalyst (*m*/*z* 1209, black, P-CAT); the [(L)_2_Au_2_(R1–H)]^+^ intermediate (*m*/*z* 1323, red, **I**_1_–**I**_4_), and the [(L)_2_Au_2_(R1–H)(C_6_H_6_)]^+^ intermediate after the reaction with benzene (*m*/*z* 1401, blue; P_1_ and P_2_). Adapted with permission from ref. 41. Copyright 2018, American Chemical Society.

A nice exercise in separating a multitude of possible isomeric intermediates comes from the Hashmi and Kappes groups.^41^ They have investigated gold-catalysed cycloisomerization reaction of a 1,5-bis-terminal diyne followed up by a coupling reaction with benzene (Fig. 6a).^41^ The reaction is catalysed by two gold cations and at every point several possible isomers can be formed. All the complexes along the catalytic cycle can be easily detected by ESI-MS, because they are cationic. However, it would be impossible to study this reaction just by means of ESI-MS^*n*^, because the fragmentation patterns of all the complexes will be rather similar.

Fig. 6b shows signals of individual mass-selected ions separated according to the ion mobilities of the contributing isomers. The reaction is catalysed by cationic gold and the authors used [(L)Au(C_3_H_3_)] (L = IPr, see the structure in Fig. 6a) as the precatalyst. It can be detected by ESI-MS as dimeric [(L)_2_Au_2_(C_3_H_3_)]^+^ cations. As expected, these ions are present as only one isomer and thus represented by a single peak in the mobilogram (black line in Fig. 6b).

The first family of reaction intermediates is formed by activation of the diyne by two cationic gold complexes ([(L)_2_Au_2_(R1–H)]^+^, see complexes labelled by a red label in Fig. 6a). The authors detected four of these intermediates (red line in Fig. 6b). The experiments can provide collision cross sections of these ions which can be compared with theoretical calculations for various possible isomers. Such analysis allowed the authors to assign the detected ions to intermediates 1–4 in Fig. 6a. The next step is a coupling between [(L)_2_Au_2_(R1–H)]^+^ and benzene. Again, a multitude of intermediates can be in principle detected (complexes labelled in blue in Fig. 6a). However, only two complexes corresponding to these intermediates were trapped by ESI-MS (blue line in Fig. 6b). Comparison of experimental and theoretical cross sections suggested that the authors probably trapped intermediates B and C (see Fig. 6a).

Hence, the authors detected intermediates along both reaction pathways as shown in Fig. 6a. Here, both pathways lead to the same product, hence the branching between the pathways does not affect the outcome of the reaction. However, in other cases, branching of reaction pathways can affect reaction selectivity. This ESI-IMS-MS approach offers an easy tool to check how reaction conditions affect the branching between the reaction pathways which might facilitate reaction optimization.

**Fig. 7:**
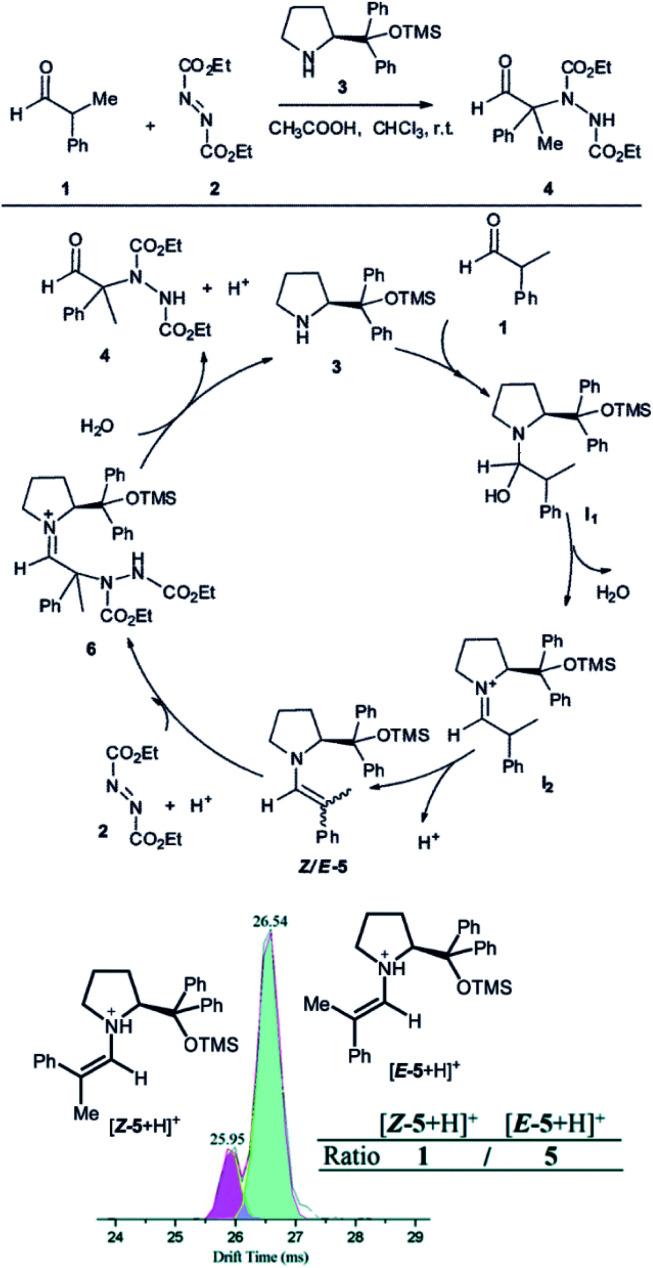
Organocatalytic reaction for C–N bond coupling. The key reactive enamine intermediate **5** was detected by ESI-MS and its *Z*- and *E*-isomers were separated by ion mobility. Reproduced from ref. 42 with permission from The Royal Society of Chemistry, Copyright 2016.

Another example, how ion mobility separation can add another dimension to mass-spectrometric investigation of reaction intermediates comes from the group of Guo.^42^ They studied intermediates formed in an organocatalytic reaction developed for C–N couplings.^1^ This type of reactions is well suited for mass spectrometry investigation, because the intermediates are either charged iminium ions (*e.g.*, **I2** in Fig. 7) or easily protonated amines or enamines (*e.g.*, **5** in Fig. 7).^43^ The organocatalytic reactions are often used for stereoselective reactions.^44^ Here, product **4** is formed as *R* or *S* stereoisomer at the α-carbon atom of the aldehyde. The stereochemistry is predetermined by the configuration of the key enamine intermediate **5**.^1^ The key finding of this study is that the ratio of enamine intermediates **Z-5** and **E-5** can be easily determined by ESI-IMS-MS approach which the authors verified by NMR spectroscopy. ESI-IMS-MS detection could be in this case an alternative to NMR or chromatography detection of these intermediates. More importantly, it can be the tool of choice for a shorter-lived intermediates that cannot be easily detected by NMR or chromatography.

**Fig. 8:**
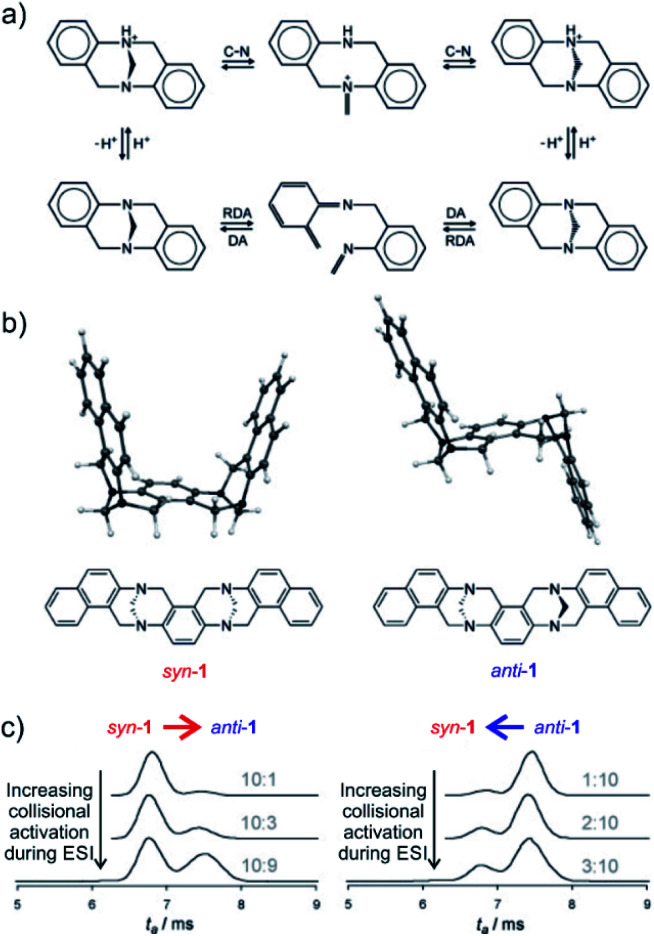
(a) Proposed pseudo-epimerization mechanisms of Tröger bases *via* iminium ions (top) and *via* retro-Diels–Alder sequence (bottom). (b) Bis-Tröger bases *syn*-**1** and *anti*-**1** and their structures predicted by DFT theory. (c) Ion-mobility traces of mass selected *syn*-**1**H^+^ (left) and *anti*-**1**H^+^ (right) as a function of the increasing cone voltage. The grey numbers are the ratios of integrated areas of the peaks corresponding to *syn*-**1** and *anti*-**1**, respectively. Adapted with permission from ref. 45. Copyright 2011 Wiley-VCH Verlag GmbH & Co. KGaA, Weinheim.

The final example showing a great promise of ion mobility separation for investigation of reaction intermediates and reaction mechanism is from the group of Schröder.^45^ They have used ESI-IMS-MS to study epimerization of a bis-Tröger bases (Fig. 8). Tröger bases are trapped as enantiomers due to the steric strain that hinders epimerization by inversion at the nitrogen atoms. Yet, these bases can epimerize by bond-breaking processes which could either correspond to formation of iminium ions upon protonation or to retro Diels Alder reaction (Fig. 8a).

The bis-Tröger base **1** can have either *syn* or *anti* configuration (Fig. 8b). The diastereoisomers *syn*-**1** and *anti*-**1** are stable and can be separated by chromatography. Electrospray ionization of these bases yields protonated ions *syn*-**1**H^+^ and *anti*-**1**H^+^. The *anti*-isomer has a more extended molecular shape which correlates with a larger collisional cross section and thus with a longer arrival time in the ion mobility experiment (Fig. 8c). The researchers used variation of electrospray ionization condition to study the pseudo-epimerization process. With increasing collisional activation during ESI, they observed increasingly epimerization of the starting isomer to the other isomer. The *syn* → *anti* isomerization required less energy than the reverse process suggesting that the *anti*-isomer is thermodynamically more stable.

The researchers also studied sodiated ions *syn*-**1**Na^+^ and *anti*-**1**Na^+^ in analogous experiments. However, these ions did not show any isomerization although the retro-Diels–Alder pathway should be accessible. Hence, this study clearly showed that pseudo-epimerization can efficiently proceed upon protonation *via* the iminium ion formation, whereas the retro-Diels–Alder pathway is mechanistically disfavoured scenario.

### Beyond mass analysis: ion spectroscopy

Ion spectroscopy is another technique that adds more dimensions to mass spectrometric separation.^46–48^ Ion spectroscopy provides IR, Vis and UV spectra of mass-selected ions, hence it adds information about their molecular and electronic structure. Small densities of mass-selected ions in a mass spectrometer do not usually permit direct measurements of absorbance/transmittance as done in classical optical spectroscopies. Instead, the absorption of photons is detected indirectly by monitoring ion fragmentation caused by photon absorption. That is why ion spectroscopy is often referred to as photodissociation spectroscopy. See recent reviews for further details.^49–51^

IR and UV/Vis spectra of mass selected ions are usually assigned based on theoretical calculations. The fact that the spectra belong to isolated ions in “vacuum” simplifies the theoretical calculations and results are often in excellent agreements. Having access to this spectroscopic information solves the problem with mass overlap for detected intermediates. In addition, it provides important structural information and thus gives deeper information about the nature of the intermediates.

**Fig. 9:**
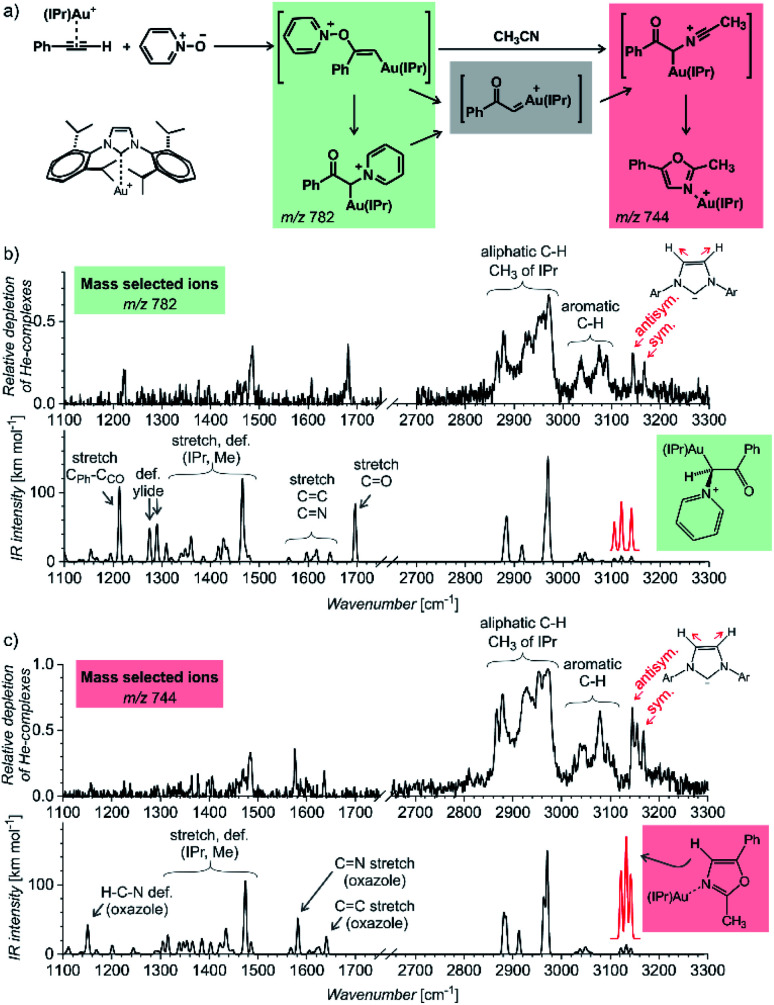
(a) Gold-mediated oxidation of phenylacetylene by pyridine *N*-oxide in the presence of acetonitrile. (b and c) IR spectra of ions with (b) *m*/*z* 782 and (c) *m*/*z* 744 measured by the helium-tagging photodissociation method^53^ and their comparison with DFT predicted IR spectra of the product complexes.^52^ Adapted with permission from ref. 52. Copyright 2016 Wiley-VCH Verlag GmbH & Co. KGaA, Weinheim.

The first example of using IR photodissociation spectroscopy for characterization of reaction intermediates is from gold chemistry.^52^ Gold activated alkynes can be oxidized by an oxygen-transfer reagents such as pyridine *N*-oxide (Fig. 9a). It has been postulated that the oxidation leads to elusive gold(i) α-oxocarbenes that react further. If reaction proceeds in acetonitrile, the reaction continues to oxazole products (see Fig. 9a). Electrospray ionization of the reaction mixture provides ions with *m*/*z* 782 and *m*/*z* 744 (among others). These ions can correspond to reaction intermediates (upper structures in the coloured boxes in Fig. 9a). The ions with *m*/*z* 782 can also represent an isomer of the primary intermediate (lower structure in the green box in Fig. 9a). This isomer can be protodeaurated to yield α-pyridinium ketone – a by-product in the reaction. The ions with *m*/*z* 744 can also correspond to the complexes between the oxazole product and the gold catalyst (the lower structure in the red box in Fig. 9a).

The IR spectra of the detected ions show that none of them corresponds to the primary intermediates (Fig. 9b and c). The spectrum of the gold complex of α-pyridinium ketone clearly displays C=O stretching vibration of the ketone function (Fig. 9b), whereas the spectrum of the gold complex of oxazole features C=N and C=C stretching bands of the oxazole ring. This simple example shows that IR photodissociation spectroscopy can provide a direct link to the structure of the detected ions and thus assist in the correct evaluation of the results (see also discussion in the next chapter).

**Fig. 10:**
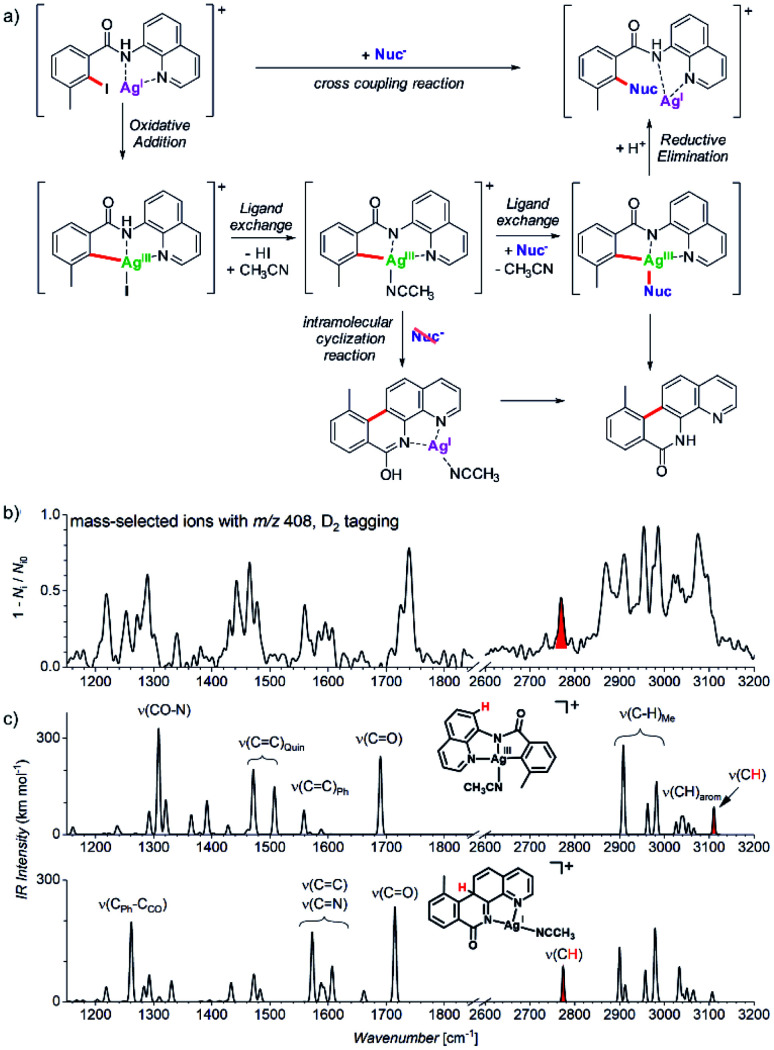
(a) Silver catalysed C–Nuc coupling and the mechanism proposed based on ESI-MS and ion spectroscopy study. (b) IR spectrum of ions with *m*/*z* 408 isolated from the reaction mixture measured by *D*_2_-tagging photodissociation method.^55,56^ (c) DFT predicted spectra for possible isomers of the detected intermediates with *m*/*z* 408. Adapted with permission from ref. 54. Copyright 2018, American Chemical Society.

Another example comes from the field of reaction development and concerns silver-catalysed C–O and C–N coupling reactions.^54^ The group of Ribas envisaged catalytic cycle based on silver(i) complexes. They designed a reaction starting with a silver(i) complex which undergoes oxidative addition with an aryl iodide to form a silver(iii) intermediate. This intermediate should exchange iodide by an *N*- or *O*-based nucleophile. The reaction sequence is completed by reductive elimination to form the new C–N or C–O bond, respectively (Fig. 10a). They demonstrated that the idea worked for a range of *N*- and *O*-nucleophiles for a substrate shown in Fig. 10. In order to prove that the reaction proceeds *via* the envisaged silver(iii) intermediates, they used mass spectrometry and ion spectroscopy.

ESI-MS of the reaction mixture showed ions with *m*/*z* 408 that formally corresponded to the expected silver(iii) intermediates bearing an acetonitrile molecule instead of iodine (see the central complex in Fig. 10a). However, the infrared spectrum of these ions did not correspond to the expected silver(iii) complex (Fig. 10b). Instead, the spectrum contained bands that could have been explained only by intramolecular cyclization of the aromatic substrate to form the tetracyclic product ions (Fig. 10c, see *e.g.*, the bands highlighted in red).

The detection of the tetracyclic product complexes of silver(i) was surprising. However, their formation can be only rationalized, if the transient silver(iii) intermediates were formed at the first place. A simple mechanistic sequence with a rollover of the quinoline group then explains the formation of the cyclized product. This initially purely mass spectrometric observation was later confirmed also synthetically. The authors performed the very same reaction without addition of the nucleophile. Indeed, they isolated the tetracyclic product as the major product of the reaction. Hence, this example shows that a detailed mass spectrometry study can not only elucidate a reaction mechanism, but it can also lead to a discovery of new reactions pathways that would be neglected otherwise.

**Fig. 11:**
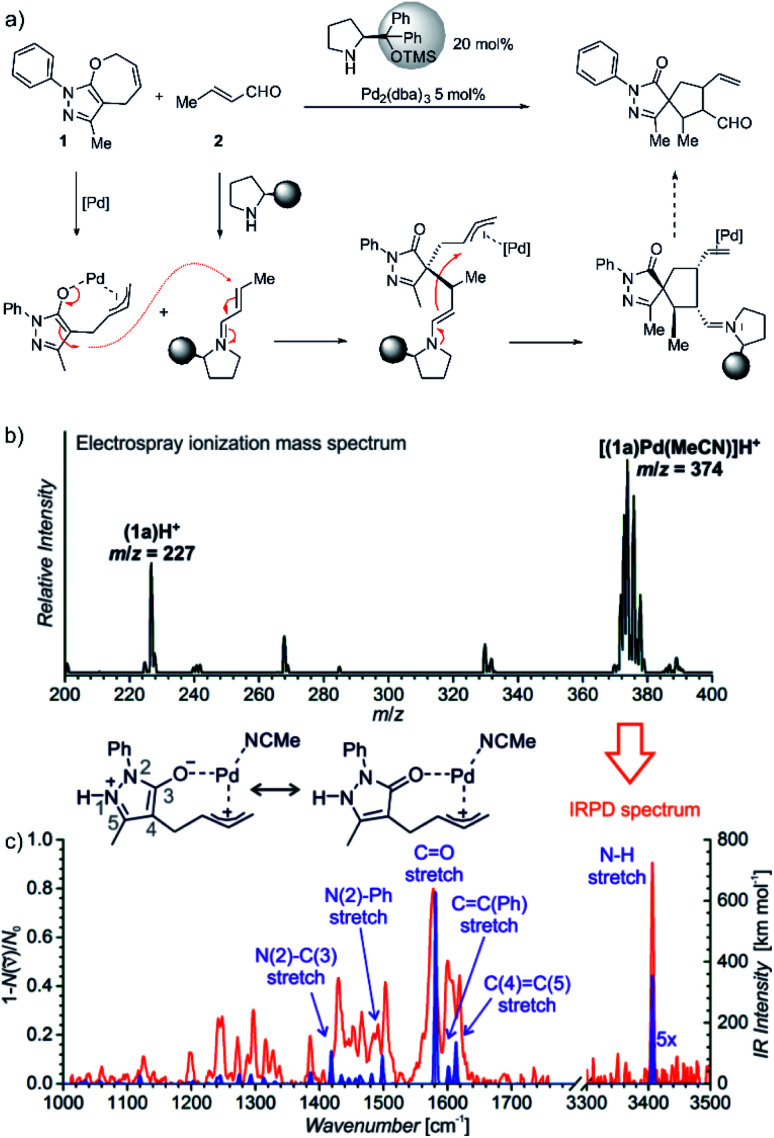
(a) Synergistic catalysis for enantioselective synthesis of spiropyrazolones and the proposed mechanism. (b) ESI-MS spectrum of 9 mM solution of **1** in CH_3_CN/CH_2_Cl_2_ (4 : 1 v/v) with 2.5 mol% Pd_2_(dba)_3_. (c) IR spectrum of [(**1**)Pd(CH_3_CN)]H^+^ (*m*/*z* 374; red line) measured by helium tagging photodissociation method and theoretically predicted spectrum (purple line) of the depicted complex. Adapted with changes from ref. 14 under CC BY 3.0 license.^26^

The last example shows how mass spectrometry and photodissociation spectroscopy can intersect the key intermediates and help in assigning the correct mechanistic pathways. The investigated reaction used palladium catalysis and organocatalysis to enable a rather complex transformation to form spiro-compounds (Fig. 11).^14^ One of the substrates was α,β-unsaturated aldehyde known to react with (chiral) pyrrolidine-based organocatalysts. The so-formed iminium intermediates readily react with nucleophiles. The second reactant (**1**) must be activated by palladium to become reactive as the nucleophile. However, the question was, how this activation happens.

Substrate **1** contains a pyrazole ring which gets easily protonated. Hence, the mass spectrometry analysis relied on charging by protonation of the substrate rather than designing a charge-tagged reactants. Accordingly, the electrospray ionization of the substrate (**1**) and the palladium catalyst led to detection of **1**H^+^ and of the protonated complex between **1** and palladium (Fig. 11).

Infrared spectrum of the mass selected palladium complex clearly revealed that the activation of **1** proceeds *via* opening of the seven-membered ring. The intermediate corresponds to a palladium-stabilized zwitter-ion. The negative charge is localized at the oxygen atom and at the pyrazole ring where the intermediate gets protonated. The structure of the protonated intermediate could be assigned based on excellent agreement between the theoretical prediction of the IR spectrum of the given complex and the experimental IR spectrum.

Knowing the structure of the intermediate, the mechanism could be rationalized. The negatively charged part of the palladium intermediate couples with the activated α,β-unsaturated aldehyde while palladium(0) stabilizes the allyl-cation fragment. The following step corresponds to the nucleophilic attack at the allyl cation.

These examples showed, how ion spectroscopy can nail down the structures of the mass-spectrometry detected intermediates. The first two examples showed that the detected ions corresponded to the product complexes. We would like to stress that this is often the case in mass spectrometric investigation of reaction mechanisms. However, we hope that the examples can convince you that even if the ions are the product ions, their detection can help in resolving the reaction mechanism or they can attest indirectly the nature of the intermediate.

### Linking mass spectra with solution chemistry

A frequent criticism of the mass spectrometry investigation of reaction intermediates and reaction mechanisms evolves from the fact that the studied processes commonly proceed in solution, whereas the detection is based on the isolated species in the gas phase. This is certainly a problem that must be well addressed in each mass spectrometric study addressing solution chemistry. The problems that occurs are:

(1) Electrospray ionization process is associated with large concentration changes. Hence, ESI-MS can detect formation of larger clusters which are artefacts of the ESI process and do not originate from solutions. Increasing the concentration can also substantially accelerate some reactions.^13,44,57^ Hence, processes that are important at high concentrations can be selectively detected, although they do not play a role at standard conditions in solution. The concentration of the solution during the spray process accelerates all condensation reactions and it has been even tested as an alternative approach for bulk synthesis.^58–60^

(2) Electrospray ionization generates ions in the field of 3–5 kV. This can lead to formation of ions that are not present in solution. For example, palladium catalysed reactions often proceed in neutral palladium complexes. However, using electrospray ionization the ionized species can be detected either because of the anionic ligand loss or because of protonation.^14^ The detection of the ionized complexes can still report about the reaction mechanism or the intermediates, but one has to be aware of the possible effect of ionization. It is also important to keep in mind that the electric field and the concentration changes can lead to the formation of species that do not have anything in common with the solution chemistry. For example, palladium(0) complexes can be oxidized in the high field to palladium(i) complexes that do not exist in solution.

(3) The ion response in electrospray ionization is not linear.^61^ Therefore, the signal intensities cannot be generally correlated with concentrations. However, the relative changes of the signal intensities can be often related qualitatively to the relative changes of concentrations of the given ions in solution. This is frequently applied in reaction monitoring using ESI-MS (see below).

(4) Ion suppression is another problem that can affect the ESI-MS detection of various species from solution. This is especially problematic if the concentration of the studied solution is above usual analytical range (<10^−5^ M), which is often the case in investigations of reaction mixtures.^62^ The ion suppression might be especially a problem, if the ionization efficiencies of reactants, intermediates and products differ. For example, formation of an easily ionisable product can lead to an increasing suppression of signals of other ions during the reaction monitoring. Hence, time evolution of some ESI-MS signals may appear as corresponding to a reaction intermediate, but in reality it might be a by-product accumulated in solution.

Keeping all these possible complications in mind, we can use ESI-MS for reaction monitoring. Many of such studies originate from the group of McIndoe.^63,64^ We will present here an example of their study of Suzuki–Miyaura reaction. Without going into details of the reaction mechanism, we will concentrate of real time monitoring of the reaction progress with ESI-MS.^63^

The palladium-catalysed cross-coupling reactions operate in the catalytic cycles between palladium(0) complexes and palladium(ii) complexes bearing two anionic ligands. Hence, the key intermediates are neutral. The McIndoe group showed that the intermediates can be detected by using charge-tagging strategy.^65^ The permanently charged group can be attached to a phosphine ligand coordinated to palladium (*e.g.*, one of the phenyl groups in Ph_3_P ligand can be sulfonated)^66^ or to a reactant.^63^ The advantage of charge tagging is that the process of the ion transfer during electrospray is quasi-identical for all the charge tagged ions and thus it allows their simultaneous monitoring and even evaluation of the signal intensity changes during the monitoring.

**Fig. 12:**
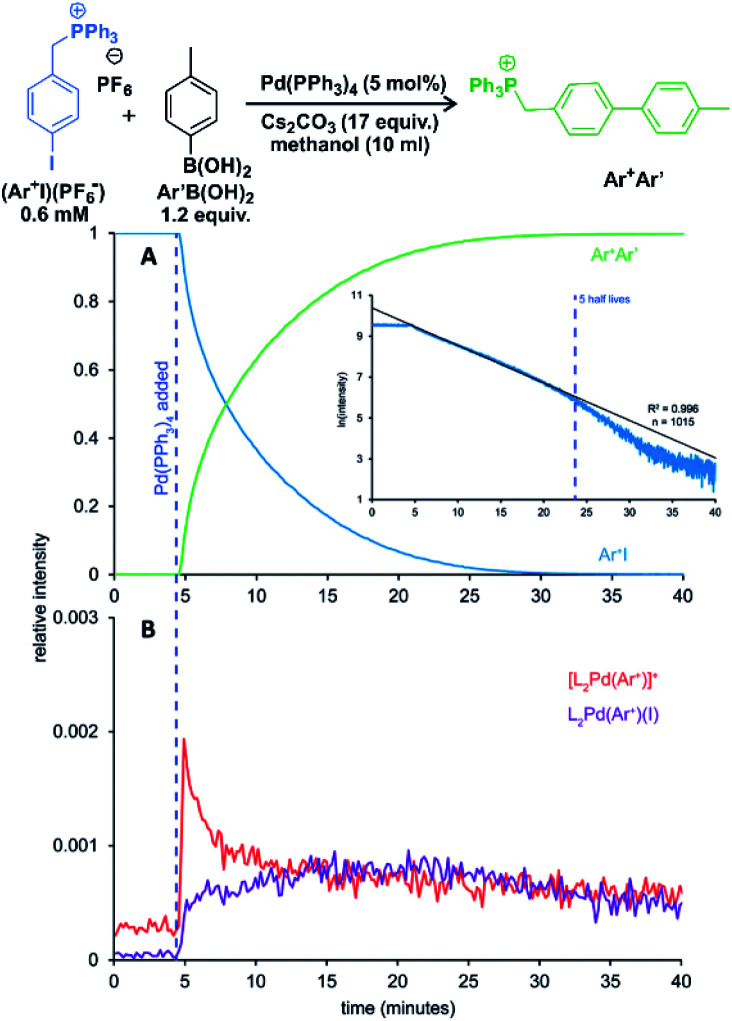
Suzuki–Miyaura coupling between a charge tagged aryl iodide (Ar^+^I) and *para*-tolyl boronic acid catalyzed by Pd(PPh_3_)_4_. (A) The ion-signal evolution of Ar^+^I and product Ar^+^Ar′. (B) Palladium intermediates. Inset: natural log of the intensity of Ar^+^I over time showing well-behaved pseudo-first-order kinetics out to 5 half-lives. Adapted with permission from ref. 63. Copyright 2018, American Chemical Society.

One of the examples of their work is investigation of transmetallation mechanisms in Suzuki–Miyaura coupling (Fig. 12).^63^ They have shown that using charge-tagging of the reactant can allow quantitative monitoring of reaction kinetics. Under the standard coupling conditions, they showed that ESI-MS data can reproduce first-order kinetics of this reaction (see the insert in Fig. 12A). They were also able to monitor the palladium containing intermediates (Fig. 12B).

In principle, it is possible to obtain quantitative information on the concentration of given species from mass spectrometry experiments. The quantification requires the use of isotopically labelled standards with known concentrations. For example, spiking of a reaction mixture with an isotopically labelled product of the given reaction could be used to quantitatively monitor the reaction progress by ESI-MS. This approach could be alternative to other means of reaction progress monitoring (*e.g.*, IR or NMR spectroscopy). However, the strength of mass spectrometry is in detecting minor reactive intermediates that cannot be addressed by other spectroscopies. For these reactive intermediates, no isotopically labelled “standards” could be prepared.

**Fig. 13:**
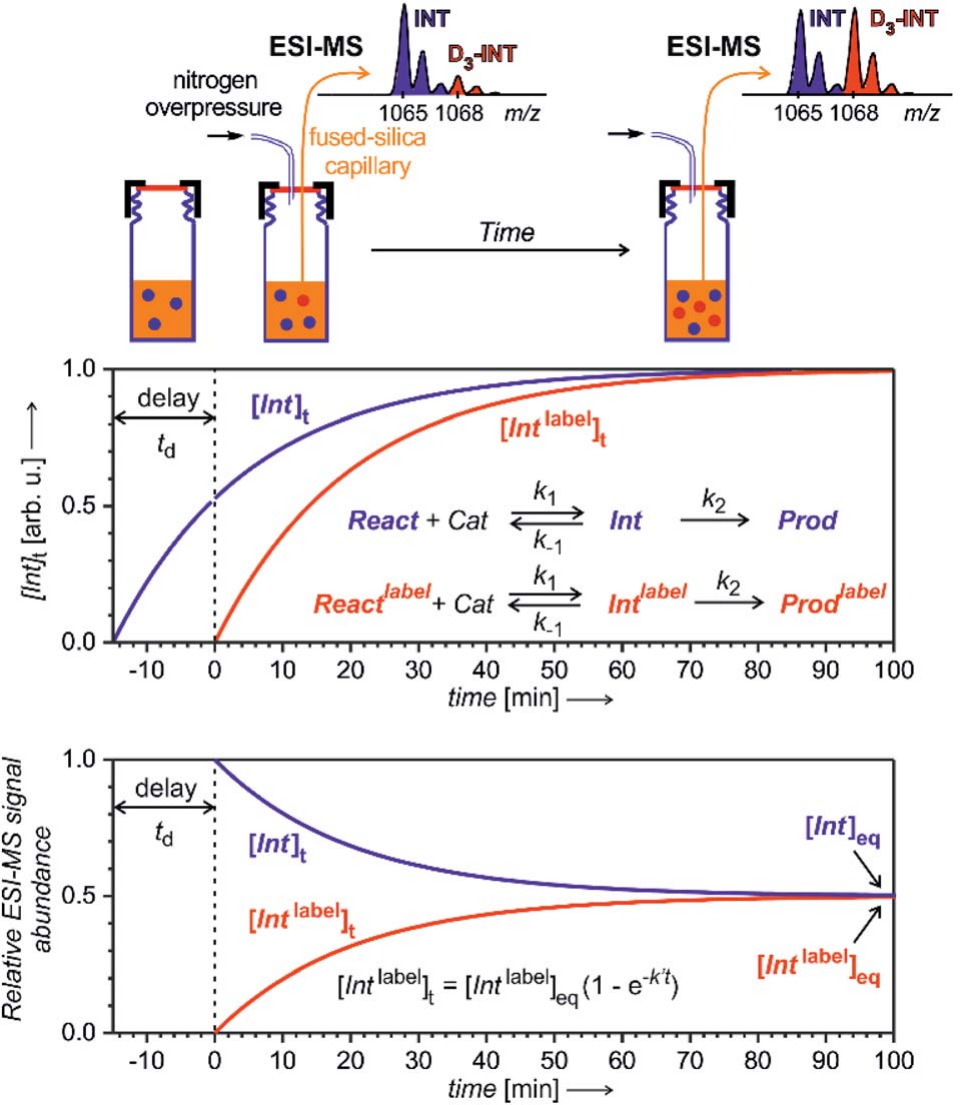
Delayed reactant labelling method for investigation of reaction intermediates formed in a steady state approximation. Delay *t*_d_ is a period, when the reaction runs only in the presence of unlabelled reactants, then an isotopically labelled reactant is added to the reaction mixture resulting in an out-of-equilibrium situation. Monitoring of the mutual evolution of the ESI-MS signals of the intermediates (bottom panel) allows determination of half-lives in steady-state approximation (see the equation: *k*′ = *k*_−1_ + *k*_2_; this particular simulation is for an intermediates with *k*′ = 0.05 min^−1^, hence with *t*_1/2_ = 14 min).

To enable quantification of reactive intermediates and to overcome the problem of non-existing isotopically labelled standards, we have designed “delayed reactant labelling” method (Fig. 13).^67^ This method is working with a reaction mixture containing isotopically labelled and unlabelled reactants that are added at a different time. The time delay introduces relative concentration differences that can be kinetically evaluated.

The example in Fig. 13 shows a reaction that proceeds *via* intermediates in steady state approximation. The reaction is started with isotopically unlabelled reactants leading to the formation of unlabelled intermediates and products. After a time delay, we add the isotopically labelled reactant allowing formation of isotopically labelled intermediates and products. Right after the mixing, the reaction mixture is out of equilibrium. The mutual evolution of the signals of the intermediates reflects the establishing of the equilibrium (Fig. 13, bottom). Similarly, the mutual evolution of products can be qualitatively evaluated, albeit it is a bit more complex (see ref. 67 for details). Note that the isotopic labelling should be remote so that no kinetic isotope effect affects the reaction (or it has to be taken into the account).

This method can be used for investigation of intermediates with half-lives on the order of minutes. If the intermediates have a very short life time, then the equilibrium is established before the reaction solution arrives at the electrospray ionization source. Hence, the signal intensities of the intermediates will only reflect the relative concentrations of the isotopically labelled and unlabelled reactants. The same is true for all “artefact” species that do not originate from solution, but are formed during the electrospray ionization process. For such species, no mutual signal intensity changes evolve.

**Fig. 14:**
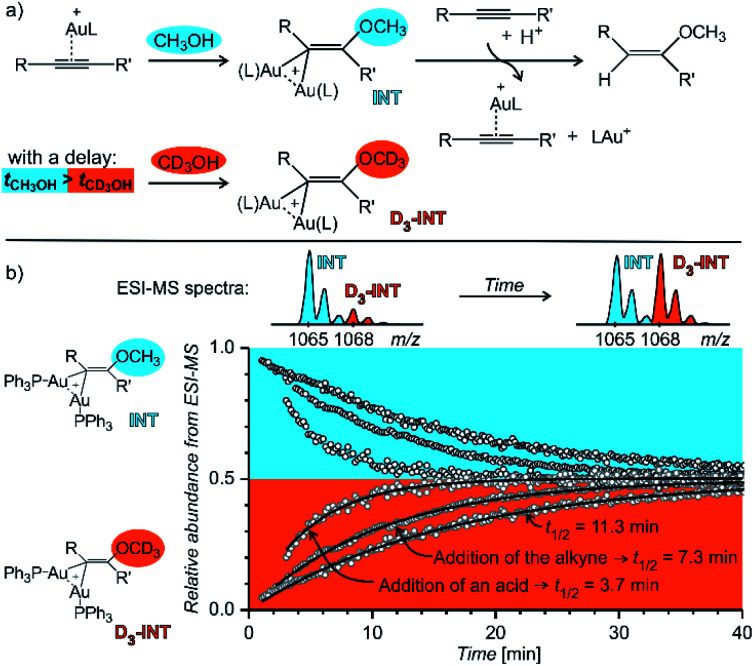
Determination of a half-lives of diaurated intermediates in gold catalysed methanol addition to alkynes. (a) Simplified reaction mechanism involving diaurated intermediates. Delayed labelling was done by diluting the reaction mixture in CH_3_OH by CD_3_OH to a double volume after 5 minutes. See the exact condition in ref. 67. (b) Mutual evolution of ESI-MS signals (open points) of isotopically unlabelled (INT) and labelled (*D*_3_-INT) intermediates. The data show acceleration of the reaction (decrease of the half-live of the intermediates) by changing of reaction conditions (increasing concentration of the alkyne and addition of *para*-toluene sulphonic acid). The half-lives were determined by fitting with the function shown in Fig. 13 (solid lines). Adapted with permission from ref. 67. Copyright 2015, American Chemical Society.

Fig. 14 shows an example of the use of delayed reactant labelling for determination of half-lives of diaurated intermediates in gold-catalysed addition of methanol to alkynes. Intermediates in this reaction can be described by the steady state approximation and their half time is ideal for mass spectrometric investigation. The changes in the half-life of the intermediates induced by changing reaction conditions directly correlate with changes of the overall reaction rate determined by NMR.^67–69^ Hence, it was possible to evaluate the effect of adding and acid, changing concentrations of reactants, effects of counter ions, and the effect of added silver salts directly on the kinetics associated with intermediates.^67–69^

**Fig. 15:**
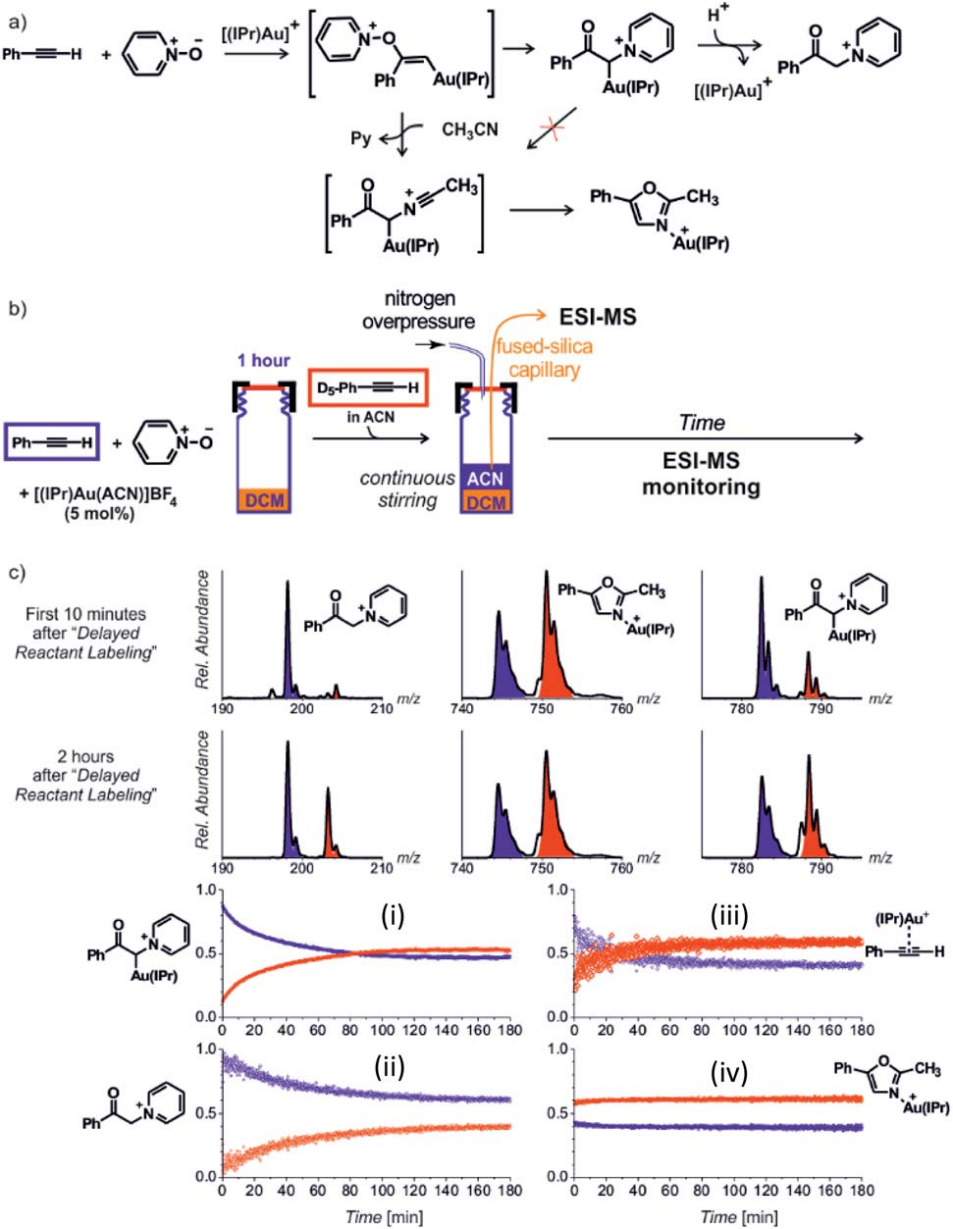
(a) Possible reaction pathways in the synthesis of oxazoline. (b) Setup for the determination of possible pathways. (c) Results of delayed reactant labelling with addition of acetonitrile only after the time delay. The opposite relative intensities of isotopically labelled and unlabelled oxazole product complex (iv) clearly shows that the reaction pathway is independent of that toward the formation of pyridinium gold-α-oxocarbenoid (i).^52^ Adapted with permission from ref. 52. Copyright 2016 Wiley-VCH Verlag GmbH & Co. KGaA, Weinheim.

Next to the quantitative evaluation of the kinetics, delayed reactant labelling can also serve to qualitatively evaluate different reaction pathways. To demonstrate this use, we wish to return to the gold mediated oxidation of alkynes shown in Fig. 9.^52^ As we have shown above, the IR photodissociation experiments revealed that the ESI-MS methods detect ions in the green and the red box in Fig. 9. The question to be resolved is, whether the pyridine group in the gold-α-oxocarbenoid intermediates could be replaced by acetonitrile (in an S_N_2 reaction) and thus the intermediates could yield the desired oxazole product (Fig. 15a).

As the reaction starts with only unlabelled reactants in dichloromethane, the reaction could only proceed *via* the pyridinium gold-α-oxocarbenoid intermediates towards the formation of α-pyridinium ketone product. After a time delay, an isotopically labelled alkyne and acetonitrile were added to the reaction mixture (Fig. 15b). The mutual evolution of the signals (Fig. 15c) of the pyridinium gold-α-oxocarbenoid intermediates (i) attest the initial larger concentration of the unlabelled intermediate is solution (the blue curve) and it shows that this intermediate in a rather slow reaction degrades to form α-pyridinium ketone products (ii) (compare the blue curves (i) and (ii) that correspond to the degradation of the unlabelled pyridinium gold-α-oxocarbenoid intermediate to the unlabelled α-pyridinium ketone product, respectively). The reaction with the labelled alkyne is reflected in the red curves (compare the evolution of curves in Fig. 15c).

Inspection of the mutual intensities of the gold-oxazole product complexes (iv) immediately shows that the signal intensities of the unlabelled and labelled product complexes are reverse and thus these complexes are not formed by the reaction of pyridinium gold-α-oxocarbenoid intermediates (i). If the pyridinium gold-α-oxocarbenoid intermediate led to the gold-oxazole product, we would expect to observe more of the unlabelled product (in blue) in the initial reaction time instead of the labelled product (in red), which is not the case in Fig. 15c (iv). Instead, the intensities of gold–oxazole product complexes roughly mirror the mutual intensities of the gold–alkyne complexes (iii) reflecting concentrations of the alkynes in solution.

This last chapter shows that ESI-MS monitoring can be used to evaluate reaction kinetics in solution albeit it requires application of appropriate methodologies. Either, we should strive to make the ionization process for the detected species as uniform as possible which can be achieved by charge-tagging. Or, we have to use relative determination of concentrations by spiking the reaction mixture with isotopically labelled standards. In the special case of the reactive species, these standards could only be formed *in situ* and we are thus left to work with relative signals as in the presented delayed reactant labelling method.

## Conclusions and outlook

Electrospray ionization provides a link between solution and mass spectrometry detection. This perspective shows how this link can be utilized to directly monitor reactions in solution. Mass spectrometry has unique sensitivity and therefore it could help in detecting key reaction intermediates or other low abundant species from solution. The trade-off is that the detection is not quantitative and that the solution-gas phase transfer might create artefacts. This perspective explains these difficulties and gives guidelines how to overcome them.

Mass spectrometric detection provides an information about the mass and thus about the elemental composition of the detected ions. However, the mere mass of the detected ions cannot be taken as a proof of detecting a particular species from solution. We discuss, how to use collision induced dissociation, ion mobility and ion spectroscopy to obtain information about the structures of the detected ions. We show that the knowledge of the structure of the detected ions is a key in evaluation of the relevance of the detected species for the solution chemistry and for the investigated reaction mechanism.

We have presented examples how ESI-MS can contribute to investigation of the key reaction steps or how it can track reaction pathways. However, we believe that ESI-MS could go even further and it could be used for discovery of new reactions. The sensitivity of ESI-MS could be employed in testing ideas for new chemical conversions. Especially, coupling with electrochemical and photochemical setups may open a door for exploration of new chemistry.

## Conflicts of interest

There are no conflicts to declare.
